# Brain re-expansion predict the recurrence of unilateral CSDH: A clinical grading system

**DOI:** 10.3389/fneur.2022.908151

**Published:** 2022-09-28

**Authors:** Shuai Han, Yan Feng, Chuanna Xu, Xuezhen Li, Fulei Zhu, Zean Li, Chunyun Zhang, Li Bie

**Affiliations:** ^1^Department of Neurosurgery of the First Clinical Hospital, Jilin University, Changchun, China; ^2^Department of Radiology of the First Clinical Hospital, Jilin University, Changchun, China

**Keywords:** chronic subdural hematoma, recurrence, computed tomography, risk factor, brain re-expansion

## Abstract

**Objective:**

Assessing the risk of postoperative recurrence of chronic subdural hematoma (CSDH) is a clinical focus. To screen the main factors associated with the perioperative hematoma recurrence. The brain re-expansion is the core factor of recurrence. A clinical prognostic scoring system was also proposed.

**Methods:**

We included 295 patients with unilateral CSDH as the training group for modeling. Factors predicting postoperative recurrence requiring reoperation (RrR) were determined using univariate and multivariate regression analyses, and bivariate Pearson correlation coefficient analysis was used to exclude related factors. Receiver operating characteristic curve analysis evaluates the ability of main factors to predict RrR and determines the cut-off value of brain re-expansion rate. We developed a prognostic scoring system and conducted preliminary verification. A verification group including 119 patients with unilateral CSDH was used to verify the grading systems.

**Results:**

The key factors for predicting unilateral CSDH recurrence were cerebral re-expansion rate (≤ 40%) at postoperative days 7–9 (OR 25.91, *p* < 0.001) and the preoperative CT density classification (isodense or hyperdense, or separated or laminar types) (OR 8.19, *p* = 0.007). Cerebral atrophy played a key role in brain re-expansion (OR 2.36, *p* = 0.002). The CSDH prognostic grading system ranged from 0 to 3. An increased score was associated with a more accurate progressive increase in the RrR rate (AUC = 0.856).

**Conclusions:**

Our prognostic grading system could screen clinically high-risk RrR patients with unilateral CSDH. However, increased attention should be paid to brain re-expansion rate after surgery in patients with CSDH.

## Highlights

- Recurrence of chronic subdural hematoma (CSDH) is the focus of clinical research.
- The brain re-expansion is the core factor of recurrence of CSDH.
- A clinical grading system was developed to rapidly and effectively predict recurrence of unilateral CSDH.

## Introduction

Chronic subdural hematoma (CSDH) is a common neurological disease in the elderly. The incidence among people over 65 years of age is 80.1/100,000/year (1), and the average age of onset is 76.8 years (2). Currently, surgery is the chief mode of treatment along with the use of drugs as a supplement. Minimally invasive surgery can quickly and effectively remove the hematoma, relieve the pressure on the brain tissue, and improve the clinical symptoms of the patient. Improved surgical skills and perioperative management have significantly reduced the postoperative recurrence requiring reoperation (RrR) rate. Nonetheless, the recurrence rate after surgery remains at 10.9–26.3% (3, 4).

Assessment of the risk of postoperative recurrence in CSDH has been proven challenging in clinical research. The risk factors of recurrence reported so far mainly include the general clinical characteristics of the patient and the surgical methods, perioperative management methods, and imaging characteristics used (5–9). Among them, imaging characteristics play a very important role.

The changes in perioperative imaging in CSDH include the following: the volume and maximum width of the hematoma, the volume and maximum width of the effusion, the distance of the midline shift, the volume of gas, the computed tomography (CT) density of the hematoma, and the effusion or signal manifestation of the MRI image of the hematoma and effusion. Considering the simple operation and relatively low cost of CT, it is commonly used for perioperative examination in clinical practice. Moreover, CT is an imaging examination and hardly involves factors affecting brain re-expansion, it is often selected as an imaging characteristic factor for predicting postoperative recurrence in CSDH patients.

This study retrospectively analyzed the general clinical characteristics and CT imaging parameters of patients with CSDH to determine the factors related to postoperative recurrence. The key factors were selected to establish a recurrence risk model grading system, which was compared with other previously published grading systems. The aim of this study is to develop a convenient and effective recurrence grading model system for clinical use.

## Methods

### Patients

A retrospective review of 295 patients with unilateral CSDH who were treated *via* surgical evacuation between July 2017 and January 2021 at The First Hospital of Jilin University was conducted. All patients were evaluated for appropriateness of surgical intervention using CT. The patients underwent burr-hole trepanation and irrigation under general anesthesia, following which a catheter attached to a closed-system drainage was constructed. The drainage tube of all patients with CSDH was removed within 24 h after the surgery. 7.5% (22/295) of patients received antiplatelet therapy and 2.4% (7/295) received anticoagulant therapy.

The following clinical and demographic data were recorded: sex, age, atrophy, history of trauma, smoking, alcohol abuse, comorbidities (hypertension, diabetes mellitus, heart disease, and cerebral infarction), anticoagulant and antiplatelet use, coagulation evaluation (platelet count, INR, and APTT), and complication (epilepsy). These patients taking antiplatelet and anticoagulant drugs all suspended the medication 1 week before the operation. Brain atrophy was defined as a person with enlarged sulci and cistern or enlarged ventricle compared with peers.

CT scanning was performed once preoperatively and twice postoperatively (before drainage tube removal on postoperative day 1 and patient discharge on postoperative days 7–9^th^).

A picture archiving and communication system was used to gather preoperative and postoperative radiographic data, which included the preoperative hematoma characteristics (volume, density characteristics (10), and maximal thickness), postoperative effusion characteristics (volume, density characteristics, and maximal thickness), postoperative residual air volume, pre- and postoperative midline shift, and cerebral volume re-expansion rate [the equation: (preoperative hematoma volume – postoperative effusion volume)/preoperative hematoma volume × 100%] from the CT scans of the head. Quantitative imaging characteristics were analyzed using the Philips IntelliSpace Discovery 3.0 software (ISD3.0, Philips, US). For quantitative volumetric analysis, the hematoma, effusion, and residual air margins were traced for each axial slice, and the volumes were calculated using the software. The quantitative image analysis was performed by a neuroradiologist who was blinded to the CSDH recurrence.

Recurrence was defined as reoperation after the first surgery due to hematoma re-accumulation by CT scan and reappearance of neurological deficits with 6 months (exclude postoperative acute subdural hematoma). And patients taking antiplatelet and anticoagulant drugs were not included as patients who requiring reoperation.

All patients underwent a six-month follow-up. Ethical approval for this study was obtained from the Institutional Review Board of The First Hospital of Jilin University (IRB00008484). The requirement to obtain informed consent from the patients for the use of the materials was waived based on the retrospective nature of the study under the approval of the IRB.

### Statistical analyses

Research data were described using categorical variables (percentage of patients) and continuous variables (mean ± standard deviation or median + 25–75 IQR). The mean ± standard deviation and median + 25–75 IQR was respectively applied to continuous variables that conform to the normal distribution and not conform to the normal distribution. The risk factors of recurrence were first performed by univariate analysis. The χ*2* test or Fisher's exact test was used for categorical variables, and the Student's *t –* test or Man-Whitney *U* test was used for continuous variables, and bivariate Pearson correlation coefficient analysis was used to exclude related factors. Subsequently, a step – wise logistical regression model was used for multivariate analysis, and variable selection was based on a *p* value of < 0.1. Regression coefficients were scaled and rounded off to obtain the weighting of variables, which provides a weight basis for the development of a prognostic system. The ability of predicting the postoperative recurrence of CSDH was determined using the receiver operating characteristic (ROC) curve. The cut-off value was defined as the highest sum of sensitivity and specificity calculated based on the Youden index. Finally, the scoring system for predicting the recurrence of CSDH was internally verified. All data were analyzed using the SPSS version 22.0 software (IBM, Armonk, New York) and the MedCalc version 19.0 software (MedCalc Software, Ostend, Belgium). A two – tailed *p* < 0.05 was considered statistically significant.

## Results

### Clinical characteristics of the patients

Of the 295 patients who underwent surgery for unilateral CSDH, 255 were males and 40 were females (age range, 21–91 years; mean age, 65.0 ± 14.0 years). Patient demographic and clinical data were shown in Table 1. Reoperation was performed on 19 patients (6.4%) (Supplementary Table 1).

**Table 1:** Clinical characteristics of patients with CSDH (*n* = 295).

**Factors**	**Number of patients (%)**		**Univariable analysis *p*-value**
	**No recurrence**	**Recurrence**	
Total	276 (93.6)	19 (6.4)	*NA*
Sex (male)	238 (86.2)	17 (89.5)	0.690
Age >65 years	154 (55.8)	16 (84.2)	0.015*
Atrophy	80 (29.0)	11 (57.9)	0.008*
History of trauma	171 (62.2)	15 (78.9)	0.143
Smoking	114 (41.3)	10 (52.6)	0.333
Alcohol abuse	60 (21.7)	6 (31.6)	0.319
Hypertension	71 (25.7)	6 (31.6)	0.574
Diabetes mellitus	58 (21.0)	6 (31.6)	0.280
Heart disease	47 (17.0)	6 (31.6)	0.110
Cerebral infarction	31 (11.2)	4 (21.1)	0.200
Anticoagulant medication	7 (2.5)	0 (0.0)	0.482
Antiplatelet medication	20 (7.2)	2 (10.5)	0.599
Platelet count < 140 × 10^3^/μL	17 (6.2)	0 (0.0)	0.265
INR > 1.2	3 (1.1)	1 (5.3)	0.235
APTT > 40 s	8 (2.9)	0 (0.0)	0.452
Postoperative epilepsy	11 (4.0)	2 (10.5)	0.179
Preoperative MGS score			0.475
0–1	209 (75.7)	13 (68.4)	
2–3	67 (24.3)	6 (31.6)	
4	0 (0.0)	0 (0.0)	
Postoperative MGS score at 1st day			0.272
0–1	242 (87.7)	15 (78.9)	
2–3	34 (12.3)	4 (21.1)	
4	0 (0.0)	0 (0.0)	
Postoperative MGS score at 7–9th day			0.179
0–1	246 (89.1)	15 (78.9)	
2–3	30 (10.9)	4 (21.1)	
4	0 (0.0)	0 (0.0)	

CSDH, chronic subdural hematoma; INR, International normalized ratio; APTT, activated partial thromboplastin time; MGS, Markwalder grading scale; NA, Not applicable.

^*^p < 0.05 was significant difference.

### Risk factors for RrR of CSDH

**Figure 1:**
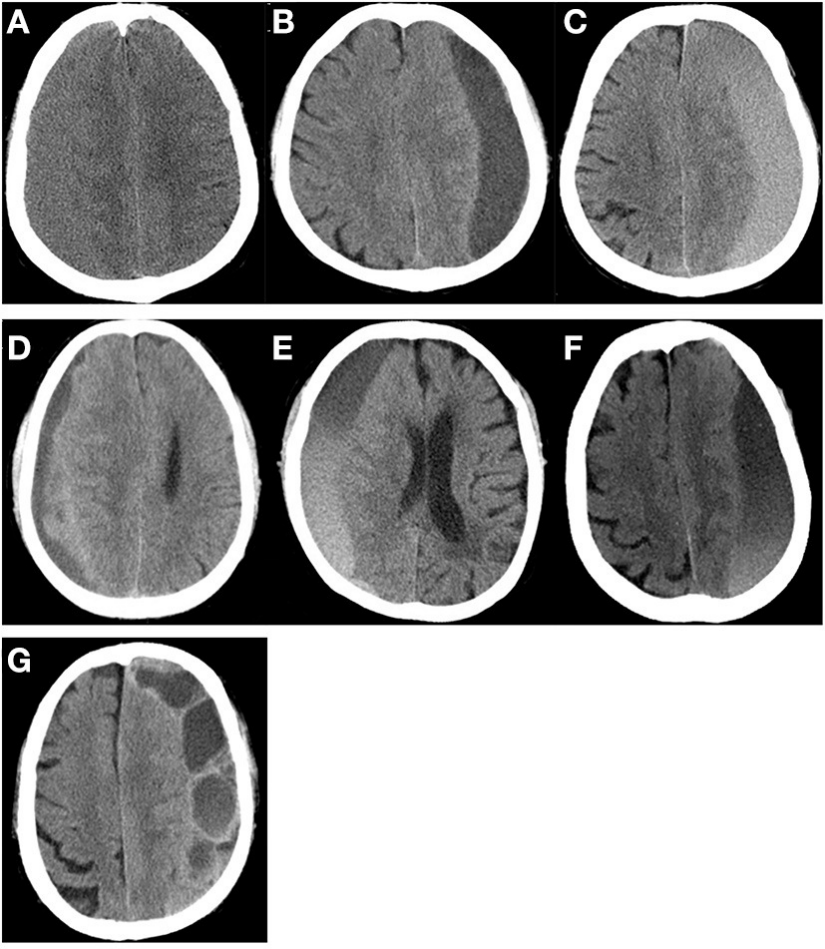
CT density classification of patients with chronic subdural hematoma. **(A)** isodense subtype, **(B)** hypodense subtype, **(C)** hyperdense subtype, **(D)** laminar type, **(E)** separated type, **(F)** gradation subtype, **(G)** trabecular type.

Associations between various individual variables and reoperation were show in Tables 1, 2. Univariate analyses showed that age >65 years, atrophy, preoperative hematoma volume, preoperative CT classification based on the density of the hematoma by Nakaguchi et al. (10) (Figure 1), and postoperative radiographic factors (effusion volume, cerebral re-expansion rate, maximal effusion thickness [> 20 mm], and midline shift [> 5 mm]) were associated with a significantly higher risk of RrR (Tables 1, 2). Furthermore, postoperative radiographic factors (effusion volume, maximal effusion thickness, midline shift, brain re-expansion rate) more sensitive on 7–9^th^ days than the preoperative day and postoperative 1^st^ day (Table 3).

**Table 2:** Imaging characteristics of patients with CSDH (*n* = 295).

**Factors**	**Number of patients (%)**		**Univariable analysis**
	**No recurrence**	**Recurrence**	***p*-value**
**Preoperative CT**			
Hematoma volume (ml)	98.48 ± 38.41	119.56 ± 41.05	0.022*
Mean hematoma density (HU)	37.32 ± 7.23	39.00 ± 6.95	0.327
Maximal hematoma thickness (> 30 mm)	115 (41.7)	12 (63.2)	0.067
Midline shift (> 10 mm)	84 (30.4)	9 (47.4)	0.124
Hematoma density changes based on CT^a^	171 (62.0)	17 (89.5)	0.016*
Isodense or hyperdense subtypes and laminar or separated types			
Hypodense or gradation subtypes and trabecular type			
**Postoperative CT at 1st day**			
Effusion volume (ml)	39.84 ± 24.02	64.56 ± 35.96	0.001*
Volume re-expansion rate^b^ (%)	58.99 ± 20.01	46.30 ± 23.10	0.009*
Mean effusion density (HU)	18.69 ± 7.83	16.47 ± 5.95	0.227
Maximal effusion thickness (> 20 mm)	53 (19.2)	10 (52.6)	0.001*
Thickness re-expansion rate^c^ (%)	45.98 ± 15.12	38.39 ± 14.98	0.035*
Midline shift (> 5 mm)	66 (23.9)	11 (57.9)	0.001*
Midline re-expansion rate^d^ (%)	71.1 ± 31.66	52.08 ± 27.94	0.010*
Gas volume (> 10 ml)	50 (18.1)	5 (26.3)	0.376
**Postoperative CT at 7–9th day**			
Effusion volume (ml)	39.62 ± 23.26	76.49 ± 35.78	< 0.001*
Volume re-expansion rate^b^ (%)	59.23 ± 20.75	33.07 ± 17.35	< 0.001*
Mean effusion density (HU)	19.50 ± 6.31	21.16 ± 7.50	0.274
Maximal effusion thickness (> 20 mm)	45 (16.3)	11 (57.9)	< 0.001*
Thickness re-expansion rate^c^ (%)	49.33 ± 13.66	33.03 ± 12.17	< 0.001*
Midline shift (> 5 mm)	37 (13.4)	7 (36.8)	0.006*
Midline re-expansion rate^d^ (%)	82.25 ± 25.89	49.31 ± 32.84	< 0.001*
Gas volume (> 10 ml)	12 (4.3)	1 (5.5)	0.851

CSDH, chronic subdural hematoma.

^a^Nakaguchi classification of chronic subdural hematoma, ^b^Volume re-expansion rate = (preoperative hematoma volume–postoperative effusion volume) /preoperative hematoma volume × 100%, ^c^Thickness re-expansion rate = (pre maximal thickness–post maximal thickness) / pre maximal thickness × 100%, ^d^Midline re-expansion rate = (pre midline shift–post midline shift) / pre midline shift × 100%.

^*^p < 0.05 was significant difference.

**Table 3:** Comparison of radiological characteristics at different times (*n* = 295).

**Radiological factors**	**Preoperative day**		**Postoperative 1st day**		**Postoperative 7**–**9th day**		***p*** **-value**		
	**AUC**	**95% CI**	**AUC**	**95% CI**	**AUC**	**95% CI**	**pre vs. 1st**	**pre vs. 7–9th**	**1st vs. 7–9th**
Hematoma (effusion) volume (ml)	0.663	0.606–0.717	0.723	0.669–0.774	0.848	0.802–0.887	0.358	0.0001*	0.129
Maximal hematoma (effusion) thickness (mm)	0.648	0.590–0.702	0.746	0.693–0.795	0.856	0.811–0.894	0.048	< 0.0001*	0.007*
Midline shift (mm)	0.655	0.597–0.709	0.707	0.651–0.758	0.791	0.740–0.836	0.285	0.053	0.183
Mean hematoma (effusion) density (HU)	0.585	0.527–0.642	0.584	0.525–0.641	0.575	0.516–0.632	0.987	0.912	0.933
Gas volume (ml)	*NA*	*NA*	0.505	0.447–0.564	0.509	0.450–0.567	*NA*	*NA*	0.943
Volume re-expansion rate (%)	*NA*	*NA*	0.678	0.622–0.731	0.840	0.793–0.880	*NA*	*NA*	0.084
Thickness re-expansion rate (%)	*NA*	*NA*	0.644	0.587–0.699	0.823	0.774–0.865	*NA*	*NA*	0.002*
Midline re-expansion rate (%)	*NA*	*NA*	0.670	0.613–0.723	0.781	0.730–0.827	*NA*	*NA*	0.051

AUC, the areas under the curve; CI, confidence interval; pre, preoperative; NA, Not applicable.

^*^p < 0.05.

### Predicting the formula of cerebral re-expansion

**Figure 2:**
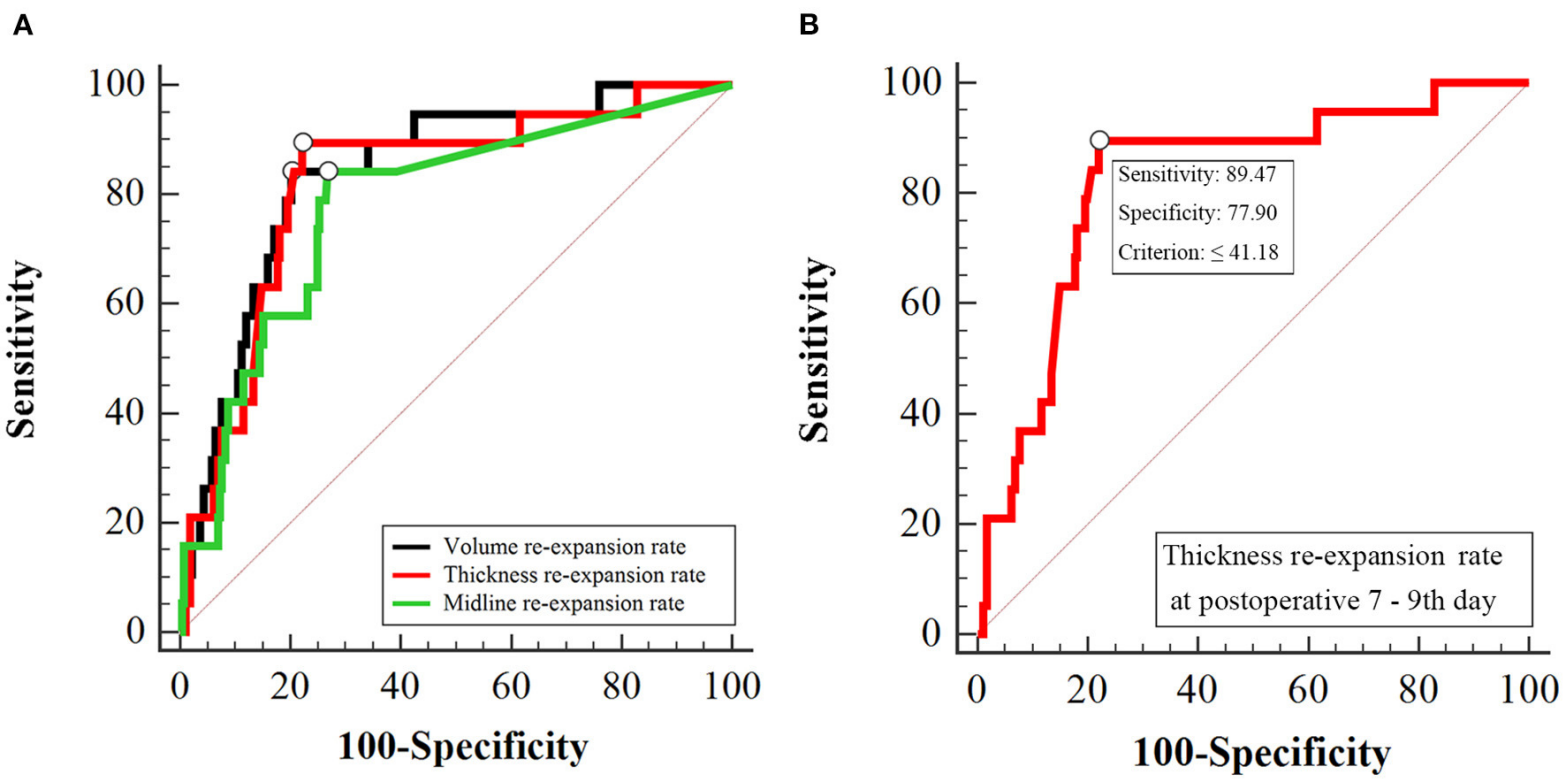
Receiver operating characteristic curve analysis for postoperative brain re-expansion to predict CSDH recurrence. **(A)** Comparison of the three different of re-expansion at postoperative 7–9th day. The area under the curve of volume re-expansion rate, thickness re-expansion rate and midline re-expansion rate are 0.840, 0.823, 0.781, respectively (*p* > 0.05). **(B)** Cut-off value of thickness re-expansion rate at postoperative 7–9th day. The cut-off value of the thickness re-expansion rate at postoperative 7–9th day is 41.18%, sensitivity is 89.47%, specificity is 77.90%.

In previous studies, the cerebral re-expansion rate was determined by calculating the change in the maximum thickness or volume of hematoma before and after surgery (Supplementary Table 2). In addition, we proposed that the midline shift could reflect the cerebral re-expansion rate before and after surgery. ROC curve analysis was used to compare the abilities of three cerebral re-expansion formulas to predict RrR (AUC 0.840, 0.823, 0.781, *p* > 0.05). Pairwise comparisons of the AUCs of the three cerebral expansion formulas did not show any statistically significant differences (Figure 2). Moreover, the maximum thickness re-expansion rate was clinically easy to calculate and did not require any other software. The cerebral re-expansion rate was used based on the maximum thickness for the analysis of the clinical data in this study.

### Risk factors for cerebral re-expansion

The factors that affected the cerebral re-expansion rate were evaluated. The cut-off point of cerebral re-expansion rate determined by ROC curve analysis was 41.18% (Figure 2). Re-expansion rates of >40 and ≤ 40% were considered good re-expansion and partial re-expansion, respectively. Univariate analysis showed that the cerebral re-expansion rate was significantly related to cerebral atrophy (*p* = 0.002). Multivariate analysis showed that cerebral atrophy was the only risk factor related to the cerebral re-expansion rate (OR 2.36, *p* = 0.002) (Supplementary Table 3).

### RrR grading system of CSDH

Among the CT imaging factors, the effusion volume, maximum thickness, and midline shift were related to volume, maximum thickness, and midline shift re-expansion, respectively (*r* = −0.679, −0.533, −0.903, *p* < 0.001, Supplementary Table 4). The three formulas for brain re-expansion had basically no significant difference in predicting recurrence (Figure 2). The maximum thickness re-expansion rate was convenient for clinical measurement and was selected as the representative brain re-expansion. Therefore, among these imaging factors that mainly affect RrR, only the factor of maximum thickness re-expansion rate remains. Among the main clinical risk factors, age is related to brain atrophy (*r* = 0.476, *p* < 0.001), and brain atrophy mainly affects the patient's brain re-expansion (OR 2.36, *p* = 0.002), so we excluded the two factors of age and brain atrophy. In addition, preoperative CT density classification was also a major risk factor for RrR (*p* = 0.016). The two main factors of preoperative CT density classification and postoperative maximal thickness re-expansion were included in the multivariate logistical regression analysis. Multivariate analysis showed that isodense / hyperdense or separated/laminar types of CT images (OR 8.19, *p* = 0.007) and cerebral re-expansion rate at postoperative 7–9^th^ days (OR 25.91, *p* < 0.001) were independent risk factors for potential CSDH recurrence (Table 4). These results of the regression modeling were the basis for the development of the CSDH classification system. Risk factors related to RrR were used in a statistical selection test to identify and create the effective model for a scoring system based on high-risk patient groups for RrR. The grading system developed in this study consisted of the preoperative hematoma density on CT and the re-expansion at postoperative days 7–9, and scores were assigned based on the strength and regression coefficients associated with RrR. The CSDH prognostic grading system ranged from 0 to 3 (Table 5). An increase in the score was associated with a more accurate progressive increase in the RrR rate (Nagelkerke R^2^ = 0.357, *p* < 0.001).

**Table 4:** Univariate and multivariate analysis of factors related to CSDH recurrence (*n* = 295).

**Factors**	**Univariate analysis**			**Multivariate analysis**		
	**OR**	**95% CI**	***p*-value**	**OR**	**95% CI**	***p*-value**
Density: isodense or hyperdense or separated or laminar types	5.22	1.18–23.05	0.029*	8.19	1.75–38.21	0.007*
Thickness re-expansion at postoperative 7–9th day (≤ 40%)	20.49	5.77–72.75	< 0.001*	25.91	7.11–94.35	< 0.001*

OR, odds ratio; CI, confidence interval.

^*^p < 0.05, Nagelkerke R^2^ = 0.357.

**Table 5:** Changchun CSDH grading system for prediction of RrR (*n* = 295).

**Components of the grading system (Unilateral)**				**Score points**
Preoperative hematoma density on CT				
Hypodense or gradation subtypes and trabecular type				0
Isodense or hyperdense subtypes and laminar or separated types				1
Thickness re-expansion at postoperative 7–9th day				
>40%				0
≤ 40%				2
Total score				0–3
**Total score points**	**No recurrence**	**Recurrence**	**Rate of recurrence (95% CI) (%)**	***p*** **-value**
0	76	0	0 (0.0–6.3)	< 0.001*
1	143	3	2.1 (0.3–4.4)	
2	29	2	6.5 (2.7–15.6)	
3	28	14	33.3 (18.5–48.2)	

RrR, recurrence requiring reoperation; CI, confidence interval.

^*^p < 0.05.

### Internal validation and comparison of the RrR model

Internal validation with the other cohorts (*n* = 119, Supplementary Table 4) was performed to examine the predictive power. Furthermore, the Changchun model was compared with different grading models to predict the RrR (Supplementary Table 5 and Figure 1). The results showed that the Changchun model was more accurate than the different models in its ability to predict the RrR (*p* < 0.001). Likewise, the ROC curve analysis revealed that the Changchun model was better at predicting the recurrence in Table 6 (AUC = 0.856).

**Table 6:** Comparison of the different grading systems (n = 119).

**Grading systems**	**AUC**	**95% CI**
Olso grading system (post 1st day)	0.630	0.537–0.717
Olso grading system (post 7–9th day)	0.716	0.626–0.795
Alberta grading system (pre)	0.544	0.451–0.636
Wuhu grading system (post 1st day)	0.550	0.457–0.642
Xining grading system (post 1st day)	0.571	0.477–0.662
Changchun grading system (post 1st day)	0.695	0.604–0.776
Changchun grading system (post 7–9th day)	0.856	0.780–0.914

pre, preoperative; post, postoperative; AUC, the areas under the curve; CI, confidence interval.

## Discussion

Several factors affect the recurrence of CSDH after surgery, including the general clinical characteristics of the patient, surgical skills, perioperative management, and imaging characteristics, which are closely related to the recurrence (5–9). The clinical factors related to RrR were retrospectively analyzed in this study. As reported previously, age (>65 years) was related to recurrence (11, 12). The imaging characteristics during the perioperative period play an important role in the assessment of the RrR. The univariate analysis shows that the preoperative CT classification, volume of effusion, midline shift, effusion thickness, and cerebral re-expansion rate after surgery were related to recurrence. These results are consistent with those reported previously (9, 13–15). These characteristics can be observed by analyzing the imaging parameters during the perioperative period.

However, owing to advancements in research, clinicians can reduce surgical complications by improving the surgical skills and strengthening perioperative management, yet cases of postoperative recurrence continue to be reported. We also summarized various methods clinically to minimize recurrence, but 6.4% of patients still recurred. In order to explore this situation, we conducted this research. It is found that this may be related to the structural characteristics of the brain tissue and the pathological characteristics of the hematoma.

### Cerebral re-expansion

**Figure 3:**
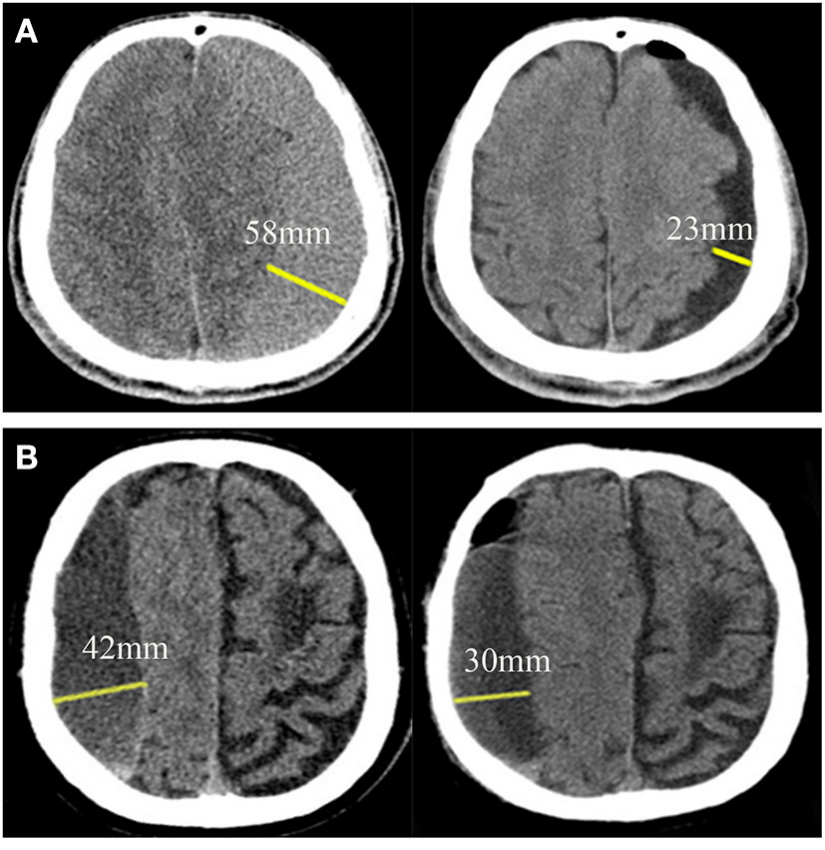
Brain re-expansion in patients with chronic subdural hematoma at postoperative 7–9^th^ day. **(A)** A patient with good brain re-expansion. The maximum thickness before surgery and 7–9 days after surgery were 58and 23 mm, respectively, and the postoperative brain re-expansion rate was 60.34%. **(B)** Another patient with partial brain re-expansion. The maximum thickness before surgery and 7–9 days after surgery were 42 and 30 mm, respectively, and the postoperative brain re-expansion rate was 28.57%.

The brain tissue is similar to an elastic sponge. A high-quality sponge has good resilience and can re-expand quickly after decompression, whereas poor quality sponges rebound slowly after compression and have poor recruitment effects (14) (Figure 3). In addition, the quality and compliance of the brain tissue of the elderly with brain atrophy are relatively poor. The cord separation of the hematoma cavity is likely to cause poor drainage. Furthermore, the presence of fresh blood in the hematoma fluid indicates that the disease is in the active phase and might be associated with postoperative recurrence (7, 16).

The quality and compliance of brain tissue can be assessed by re-expansion of brain tissue. Cerebral re-expansion rate is calculated as the change in brain tissue volume before and after surgery. Under the condition of a fixed cranial cavity volume, the effusion and volume change of a hematoma before and after surgery can indirectly reflect the cerebral re-expansion rate (17, 18). However, some studies defined brain re-expansion rate as the change in the maximal thickness of the hematoma and the maximal thickness of the effusion before and after surgery (19–21) (Supplementary Table 2). The volumes of the hematoma and the effusion in the formula for the brain re-expansion rate need to be calculated using software, which is not convenient for clinical application. In this study, three formulas were compared based on the volume, maximal thickness, and midline shift ratios. The formulas demonstrated similar predictive effects, especially the ones based on the maximal thickness ratio and the volume ratio. The calculation of the maximal thickness of the hematoma and effusion does not require software assistance and can be conveniently applied in the clinical setting. Therefore, we use the formula based on the maximal thickness ratio as that for the cerebral re-expansion rate in the grading system.

Cerebral atrophy was found to affect the re-expansion of the brain tissue. In addition, age (>65 years) and injury time (>30 days) had a tendency to influence the re-expansion. Multivariate analysis revealed that atrophy was the only factor that affected the re-expansion rate. Previous studies have found that cerebral atrophy, long injury time (>30 days), and old age are high-risk factors for CSDH recurrence, and these factors are related to the cerebral re-expansion rate (19–21). This means that the cerebral re-expansion rate is a hub in the collection of the above risk factors.

We found that the comparisons of the relationship between bilateral CSDH and cerebral re-expansion rate indicated that the postoperative re-expansion ability of bilateral CSDH was weaker (*p* = 0.032, Supplementary Table 6). This result was consistent with those reported in previous studies (18). Consequently, the unilateral grading system cannot be applied to patients with bilateral CSDH. In the future, we need develop a model to predict the recurrence of bilateral CSDH (22).

Factors such as aging-related brain atrophy, postoperative coagulation dysfunction, low intracranial pressure, and secondary injury are all related to partial postoperative cerebral re-expansion (18, 20, 23). However, partial cerebral re-expansion after evacuation resulted in prolonged subdural space enlargement, which created the possibility of hematoma re-accumulation. Hence, brain re-expansion and postoperative recurrence are closely related, but patients whose radiographic partial or non-expanded brain is not associated with neurological damage or symptoms are usually treated conservatively. Therefore, the relationship between cerebral re-expansion rate and neurological recovery still needs further research.

### Characteristics of the CT imaging

The pathological characteristics of CSDH are closely related to the characteristics of the CT imaging (10). The density of the images on the CT scan is closely related to hematoma recurrence (24). The homogeneous type includes three subtypes (hypodense, isodense, and hyperdense). The separated type is defined as a higher density component under a lower density component, and there is a clear boundary between them. If two components are mixed without a boundary, it is called the gradation type. The laminar type is defined as a hematoma that presents with a dense layer running along the inner membrane. The trabecular type is defined as a hematoma with a low iso-density component and a high-density septum that separates the inner and outer membranes. In the pathophysiology of CSDH, the hypodense and gradation subtypes are considered to have a moderate tendency to re-bleed, and the trabecular type is considered to be the regression stage of these lesions (10). The isodense, hyperdense, laminar, and separated types have a high-risk of recurrence (9). Conversely, the hypodense, gradation, and trabecular types have a low risk of recurrence.

### A clinical grading system

The data of 295 patients were used to establish a model scoring system to assess the recurrence of CSDH following which another group (119 patients, June 2015 to July 2016) was used to verify the grading system. The factors predicting postoperative recurrence were screened out, the related factors are eliminated, and those that met the criteria were incorporated into the multiple regression analysis model. It was concluded that the cerebral re-expansion rate and preoperative CT imaging classification were important independent predictors of RrR. According to the ROC curve analysis, the critical threshold of the postoperative cerebral re-expansion rate (cut-off point 40%) was determined. According to the intensity and regression coefficients associated with RrR to assign scores to establish a grading system. The prediction performance of the model was compared with those of the previously published models in the validation group, and the model had a better evaluation effect. The main parameters of the model were easy to collect clinically and could quickly screen the RrR high-risk patients, thereby providing a reference for guiding the treatment.

The parameters of postoperative day 1 in previous studies were usually used to predict and evaluate the possibility of postoperative recurrence of CSDH (22, 25). However, in the current study, the imaging parameters of postoperative day 1 were compared with those of days 7–9. The 7–9^th^ day parameters demonstrated better predictive abilities of the recurrence of CSDH (Table 3). The re-expansion is greatest during the first week after surgery and slows down considerably after that (14).

Past studies have established models to evaluate patients with high-risk RrR. They have good clinical application values but are associated with some shortcomings. The Alberta grading system only includes preoperative clinical and imaging parameters without the postoperative factors and cannot fully reflect the perioperative imaging changes (26). In the Oslo grading system, the preoperative hematoma volume cut-off point is 130 mL, and the postoperative residual cavity volume cut-off points are 80 and 120 mL (25). In the Xining grading system, the thresholds for the volume of the hematoma before the operation and the volume of the postoperative residual cavity are 121 and 72 mL, respectively (27). However, it is not suitable for different races to use the same fixed volume parameter threshold due to limitations in clinical application. The Xining grading system adopts the Nomogram Model, which is a relatively innovative method, but the outcome of predicting the recurrence is binary. The proportion of postoperative gas accumulation in the Wuhu grading system is an important factor (28). However, with improvements in surgical skills, the amount of postoperative gas produced is reduced and does not affect the patient's prognosis (29). On the contrary, the cerebral re-expansion rate is the core and central point that accurately reflects the patient's perioperative imaging changes.

### Methods to improve brain re-expansion

According to this study, in order to further reduce RrR, the main purpose is to improve brain re-expansion. The current methods used to increase the re-expansion rate of the brain tissue include the following: intraoperative aspiration of pneumocephalus *via* a subdural drain following evacuation (30), neuroendoscopic removal of the residual septa and trabecula structures to promote brain expansion (31), postoperatively performed supervised Valsalva maneuver (32), administration of at least 2,000 mL per 3 days (33), and early mobilization (34). These methods reduce potential subdural space and promote cerebral expansion, thereby decreasing RrR.

### Limitations

One of the limitations of this study is that it is a single-center study with a small sample size. Hence, further verifications using multicenter studies with a larger sample size are warranted. Although another set of data was used to verify the model system, the model did not perform prospective, multicenter verification. Therefore, it is necessary to apply the datasets collected from other centers to verify our findings in the future. In addition, many neurosurgical centers around the world don't routinely perform postoperative CT scans. In view of this situation, we may further study the preoperative predictive grading model of brain re-expansion in the future to make the research more applicable in clinical practice. The recurrence rate is relatively low because we have learned various methods to reduce the recurrence of hematoma on the basis of previous research and conducted multiple short-term clinical follow-ups. In addition, this study only involves patients with unilateral hematoma, and the risk factors for bilateral CSDH recurrence and should be further studied.

## Conclusions

In this study, cerebral re-expansion was found to play an important role in the RrR of CSDH. On the basis of the preoperative CT imaging classification and cerebral re-expansion rate, a grading model system for assessing recurrence was established. This model was validated and compared with previously used models, and it was found to be more effective. These findings suggest that the postoperative recurrence of CSDH can be effectively reduced by increasing the cerebral re-expansion rate.

## Data availability statement

The raw data cannot be obtained at the request of the hospital image database. If there is a suitable reason, the individual image data can be made accessible by contacting corresponding author LB with a research application.

## Ethics statement

The studies involving human participants were reviewed and approved by the Ethics Committee of the First Hospital of Jilin University. Written informed consent for participation was not required for this study in accordance with the national legislation and the institutional requirements.

## Author contributions

LB contributed to the study conception and design. Data analysis was performed by SH. The first draft of the manuscript was written by LB. All authors were involved in material preparation, data collection, commented on previous versions of the manuscript, read, and approved the final manuscript.

## Funding

This study was funded by National Natural Science Foundation of China (No. 81201980 and No. 81572476).

## Conflict of interest

The authors declare that the research was conducted in the absence of any commercial or financial relationships that could be construed as a potential conflict of interest.

## Publisher's note

All claims expressed in this article are solely those of the authors and do not necessarily represent those of their affiliated organizations, or those of the publisher, the editors and the reviewers. Any product that may be evaluated in this article, or claim that may be made by its manufacturer, is not guaranteed or endorsed by the publisher.
