# Supplementary material for: Brain re-expansion predict the recurrence of unilateral CSDH: A clinical grading system
Source: Front Neurol. 2022 Sep 28;13:908151. doi: 10.3389/fneur.2022.908151 (PMC9554254; doi:10.3389/fneur.2022.908151)
Supplement: Supplementary file 1 [file Table_1.docx]

| **Supplement table 1. Demographic information of the recurrence of unilateral CSDH** | | | | | | |
| --- | --- | --- | --- | --- | --- | --- |
| Group (n) | | Patients | Age (years) | Sex (F/M) | Preoperative MGS score | Number of Recurrence |
| Training dataset (n = 295) | | 1 | 66 | M | 2 | 1 |
|  |  | 2 | 67 | M | 1 | 1 |
|  |  | 3 | 76 | M | 3 | 1 |
|  |  | 4 | 77 | M | 3 | 2 |
|  |  | 5 | 75 | M | 1 | 1 |
|  |  | 6 | 65 | M | 1 | 1 |
|  |  | 7 | 68 | M | 1 | 1 |
|  |  | 8 | 70 | M | 1 | 1 |
|  |  | 9 | 66 | F | 1 | 1 |
|  |  | 10 | 51 | M | 1 | 1 |
|  |  | 11 | 73 | M | 1 | 1 |
|  |  | 12 | 69 | M | 2 | 1 |
|  |  | 13 | 91 | M | 3 | 1 |
|  |  | 14 | 74 | M | 1 | 1 |
|  |  | 15 | 61 | M | 1 | 1 |
|  |  | 16 | 70 | M | 2 | 1 |
|  |  | 17 | 78 | F | 1 | 1 |
|  |  | 18 | 67 | M | 1 | 1 |
|  |  | 19 | 57 | M | 1 | 1 |
| Testing dataset  (n =119) |  | 1 | 76 | M | 1 | 1 |
|  |  | 2 | 65 | M | 1 | 1 |
|  |  | 3 | 84 | M | 3 | 1 |
|  |  | 4 | 79 | M | 2 | 1 |
|  |  | 5 | 72 | M | 1 | 1 |
|  |  | 6 | 45 | F | 1 | 1 |
|  |  | 7 | 66 | M | 1 | 1 |
|  |  | 8 | 74 | M | 1 | 1 |
|  |  | 9 | 63 | M | 1 | 1 |
