# Supplementary material for: Brain re-expansion predict the recurrence of unilateral CSDH: A clinical grading system
Source: Front Neurol. 2022 Sep 28;13:908151. doi: 10.3389/fneur.2022.908151 (PMC9554254; doi:10.3389/fneur.2022.908151)
Supplement: Supplementary file 2 [file Table_2.docx]

| **Supplement table 2. Formula of brain re-expansion rate of published articles** | | | | | | |
| --- | --- | --- | --- | --- | --- | --- |
| Definition | Formula of re-expansion rate | Calculation basis | Patients No. | CT scan time | Authors & Year | References |
| brain re-expansion rate | (pre – post) / pre × 100% | hematoma thickness | 500 | post 7th day | Mori K et al. 2001 | [20] |
| brain re-expansion rate | (pre – post) / pre × 100% | hematoma thickness | 621 | post 7th day | Mori K et al. 2003 | [19] |
| brain re-expansion rate | (pre – post) / pre × 100% | hematoma volume | 20 | post 14th,30th day | Kung WM et al. 2012 | [15] |
| brain re-expansion rate | (pre – post) / pre × 100% | hematoma volume | 125 | post 2nd day | Jeong SI et al. 2014 | [11] |
| brain re-expansion rate | (pre – post) / pre × 100% | hematoma thickness | 130 | post 3 months | Ro HW et al. 2016 | [25] |
| pre: preoperative, post: postoperative, Patients No. : Number of patients. | | | | | |  |
