# Supplementary material for: Brain re-expansion predict the recurrence of unilateral CSDH: A clinical grading system
Source: Front Neurol. 2022 Sep 28;13:908151. doi: 10.3389/fneur.2022.908151 (PMC9554254; doi:10.3389/fneur.2022.908151)
Supplement: Supplementary file 3 [file Table_3.docx]

| **Supplement table 3. Univariate and Multivariate analysis of factors predicting cerebral re-expansion (n = 295)** | | | | | |
| --- | --- | --- | --- | --- | --- |
| Factors | Cerebral re-expansion | | Univariable analysis *p* value | Multivariable analysis *p* value | Multivariable analysis  OR (95% CI) |
|  | Good (%) | Partial (%) |  |  |  |
| Total | 222 (75.3) | 73 (24.7) | *NA* |  |  |
| Sex (male) | 195 (87.8) | 60 (82.2) | 0.222 |  |  |
| Age > 65 years | 122 (55.0) | 48 (65.8) | 0.105 |  |  |
| Atrophy | 58 (26.1) | 33 (45.2) | 0.002* | 0.002* | 2.36 (1.36 - 4.11) |
| Trauma > 30 days | 88 (39.6) | 37 (50.7) | 0.098 | 0.089 | 1.60 (0.93 - 2.75) |
| Smoking | 98 (44.1) | 26 (35.6) | 0.200 |  |  |
| Alcohol abuse | 48 (21.6) | 18 (24.7) | 0.589 |  |  |
| Hypertension | 59 (26.6) | 18 (24.7) | 0.746 |  |  |
| Diabetes | 44 (19.8) | 20 (27.4) | 0.173 |  |  |
| Heart disease | 38 (17.1) | 15 (20.5) | 0.508 |  |  |
| Cerebral infarction | 23 (10.4) | 12 (16.4) | 0.164 |  |  |
| Anticoagulant medication | 5 (2.3) | 2 (2.7) | 0.812 |  |  |
| Antiplatelet medication | 17 (7.7) | 5 (6.8) | 0.820 |  |  |
| Platelet count < 140× 10^3^/μL | 12 (5.4) | 5 (6.8) | 0.646 |  |  |
| INR > 1.2 | 3 (1.4) | 1 (1.4) | 0.991 |  |  |
| APTT > 40 sec | 8 (3.6) | 0 (0.0) | 0.207 |  |  |
| Postoperative epilepsy | 10 (4.5) | 3 (4.1) | 0.887 |  |  |
| Preoperative hematoma volume (> 100 ml) | 110 (49.5) | 36 (49.3) | 0.972 |  |  |
| Preoperative Midline shift (> 10 mm) | 70 (31.5) | 23 (31.5) | 0.997 |  |  |
| Preoperative Mean hematoma density (HU) |  |  | 0.810 |  |  |
| < 25 | 9 (4.1) | 3 (4.1) |  |  |  |
| 25 - 35 | 76 (34.2) | 22 (30.1) |  |  |  |
| > 35 | 137 (61.7) | 48 (65.8) |  |  |  |
| Preoperative hematoma density on CT |  |  | 0.204 |  |  |
| Isodense or hyperdense subtypes and laminar or separated types | 146 (65.8) | 42 (57.5) |  |  |  |
| Hypodense or gradation subtypes and trabecular type | 76 (34.2) | 31 (42.5) |  |  |  |
| Postoperative air volume (> 10 ml) |  |  |  |  |  |
| Postoperative mean hematoma density (HU) |  |  | 0.206 |  |  |
| < 25 | 183 (82.4) | 56 (76.7) |  |  |  |
| 25 - 35 | 35 (15.8) | 17 (23.3) |  |  |  |
| > 35 | 4 (1.8) | 0 (0.0) |  |  |  |
| INR: International normalized ratio, APTT: Activated partial thromboplastin time, *NA*: Not applicable, OR: odds ratio, CI: conﬁdence interval. | | | | | |
| **p <* 0.05. |  |  |  |  |  |
