# Supplementary material for: Brain re-expansion predict the recurrence of unilateral CSDH: A clinical grading system
Source: Front Neurol. 2022 Sep 28;13:908151. doi: 10.3389/fneur.2022.908151 (PMC9554254; doi:10.3389/fneur.2022.908151)
Supplement: Supplementary file 4 [file Table_4.docx]

| **Supplement table 4. Correlation of related factors at postoperative 7 - 9th day (n = 295)** | | |
| --- | --- | --- |
| Factors | postoperative 7 - 9th day | |
|  | *r* | *p* value |
| Age *vs* Atrophy | 0.476 | < 0.001* |
| Effusion volume *vs* Volume re-expansion rate | -0.679 | < 0.001* |
| Maximal effusion thickness *vs* Thickness re-expansion rate | -0.533 | < 0.001* |
| Midline shift *vs* Midline re-expansion rate | -0.903 | < 0.001* |
| Volume re-expansion rate *vs* Thickness re-expansion rate | 0.476 | < 0.001* |
| Volume re-expansion rate *vs* Midline re-expansion rate | 0.260 | < 0.001* |
| Thickness re-expansion rate *vs* Midline re-expansion rate | 0.251 | < 0.001* |
| *r*: bivariate Pearson correlation coefficient, *vs*: versus. |  |  |
| **p* < 0.05. |  |  |
