# Supplementary material for: Brain re-expansion predict the recurrence of unilateral CSDH: A clinical grading system
Source: Front Neurol. 2022 Sep 28;13:908151. doi: 10.3389/fneur.2022.908151 (PMC9554254; doi:10.3389/fneur.2022.908151)
Supplement: Supplementary file 5 [file Table_5.docx]

| **Supplement table 5. Validation of RrR grading system (n = 119)** | | | | | | |
| --- | --- | --- | --- | --- | --- | --- |
| RrR Grading system | Total score points | No Recurrence | Recurrence | | Rate of Recurrence (95% CI) (%) | *p* value |
| Oslo grading system (postoperative 1st day) | 0 | 41 | | 2 | 4.7 (0.6 - 15.8) | 0.269 |
|  | 1 - 2 | 55 | | 4 | 6.8 (1.9 - 16.5) |  |
|  | 3 - 4 | 14 | | 3 | 17.6 (3.8 - 43.4) |  |
|  | 5 | 0 | | 0 | 0.0 |  |
| Oslo grading system (postoperative 7 - 9th day) | 0 | 42 | | 1 | 2.3 (0.1 - 12.3) | 0.048* |
|  | 1 - 2 | 54 | | 4 | 6.9 (1.9 - 16.7) |  |
|  | 3 - 4 | 14 | | 4 | 22.2 (6.4 - 47.6) |  |
|  | 5 | 0 | | 0 | 0.0 |  |
| Alberta grading system (preoperative) | 0 | 46 | | 5 | 9.8 (3.3 - 21.4) | 0.589 |
|  | 1 | 44 | | 2 | 4.3 (0.5 - 14.8) |  |
|  | 2 | 20 | | 2 | 9.1 (1.1 - 29.2) |  |
|  | 3 | 0 | | 0 | 0.0 |  |
| Wuhu grading system (postoperative 1st day) | 0 - 1 | 51 | | 4 | 7.3 (2.0 - 17.6) | 0.234 |
|  | 2 | 27 | | 2 | 6.9 (0.8 - 22.8) |  |
|  | 3 | 19 | | 0 | 0.0 (0.0 - 17.6) |  |
|  | 4 | 11 | | 2 | 15.4 (1.9 - 45.4) |  |
|  | 5 - 6 | 2 | | 1 | 33.3 (0.08 - 90.6) |  |
| Xining grading system (postoperative 1st day) | ≤ 252 | 89 | | 6 | 6.3 (2.4 - 13.2) | 0.306 |
|  |  |  |  |  |  |  |
|  | > 252 | 21 | | 3 | 12.5 (2.7 - 32.4) |  |
|  |  |  |  |  |  |  |
| Changchun grading system (postoperative 1st day) | 0 | 29 | | 1 | 3.3 (0.1 - 17.2) | 0.105 |
|  | 1 | 58 | | 3 | 4.9 (1.0 - 13.7) |  |
|  | 2 | 12 | | 2 | 14.3 (1.8 - 42.8) |  |
|  | 3 | 11 | | 3 | 21.4 (4.7 - 50.8) |  |
| Changchun grading system (postoperative 7 - 9th day) | 0 | 42 | | 0 | 0.0 (0.0 - 8.4) | < 0.001* |
|  | 1 | 58 | | 3 | 4.9 (1.0 - 13.7) |  |
|  | 2 | 5 | | 2 | 28.6 (3.7 - 71.0) |  |
|  | 3 | 5 | | 4 | 44.4 (13.7 - 78.8) |  |
