# Supplementary material for: Brain re-expansion predict the recurrence of unilateral CSDH: A clinical grading system
Source: Front Neurol. 2022 Sep 28;13:908151. doi: 10.3389/fneur.2022.908151 (PMC9554254; doi:10.3389/fneur.2022.908151)
Supplement: Supplementary file 6 [file Table_6.docx]

| **Supplement table 6. Compare unilateral CSDH and bilateral CSDH re-expansion** | | | | |
| --- | --- | --- | --- | --- |
| CSDH | Thickness re-expansion at postoperative 7 - 9th day (%) | *p* value | | |
| Unilateral | 48.28 ± 14.13 | 0.032* | | |
| Bilateral | 40.05 ± 19.65 |  | | |
| *p* < 0.05 |  | |  |  |
