# Supplementary material for: Brain re-expansion predict the recurrence of unilateral CSDH: A clinical grading system
Source: Front Neurol. 2022 Sep 28;13:908151. doi: 10.3389/fneur.2022.908151 (PMC9554254; doi:10.3389/fneur.2022.908151)
Supplement: Supplementary file 7 [file Image_1.pdf]

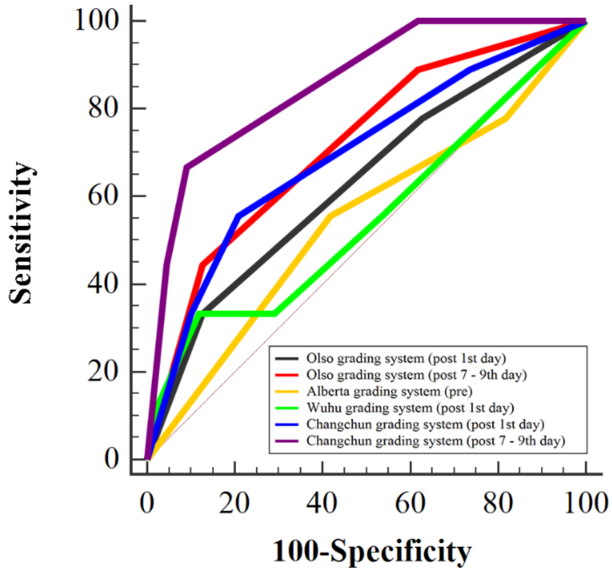

Supplement figure 1. Receiver operating characteristic curve analysis for different grading systems. the ROC curve analysis revealed that AUC area of the Changchun grading system (post 7 - 9th day) was 0.856.
